# Identification and Validation of Expressed Sequence Tags from Pigeonpea (*Cajanus cajan* L.) Root

**DOI:** 10.1155/2014/651912

**Published:** 2014-05-06

**Authors:** Ravi Ranjan Kumar, Shailesh Yadav, Shourabh Joshi, Prithviraj P. Bhandare, Vinod Kumar Patil, Pramod B. Kulkarni, Swati Sonkawade, G. R. Naik

**Affiliations:** ^1^Vidya Pratishthan's School of Biotechnology, Vidyanagari, Baramati, Pune 413133, India; ^2^Department of Biotechnology, Gulbarga University, Gulbarga, Karnataka 585106, India; ^3^Institute of Biotechnology, Acharya N. G. Ranga Agricultural University, Hyderabad 500030, India

## Abstract

Pigeonpea (*Cajanus cajan* (L) Millsp.) is an important food legume crop of rain fed agriculture in the arid and semiarid tropics of the world. It has deep and extensive root system which serves a number of important physiological and metabolic functions in plant development and growth. In order to identify genes associated with pigeonpea root, ESTs were generated from the root tissues of pigeonpea (GRG-295 genotype) by normalized cDNA library. A total of 105 high quality ESTs were generated by sequencing of 250 random clones which resulted in 72 unigenes comprising 25 contigs and 47 singlets. The ESTs were assigned to 9 functional categories on the basis of their putative function. In order to validate the possible expression of transcripts, four genes, namely, *S-adenosylmethionine synthetase*, *phosphoglycerate kinase*, *serine carboxypeptidase*, and *methionine aminopeptidase*, were further analyzed by reverse transcriptase PCR. The possible role of the identified transcripts and their functions associated with root will also be a valuable resource for the functional genomics study in legume crop.

## 1. Introduction

Pigeonpea (*Cajanus cajan* L.) Millsp. (2*n* = 22) is a major grain legume of the arid and semiarid regions of the world [1]. Though considered a minor crop, pigeonpea is of considerable importance in areas of South Asia (mainly on the Indian subcontinent), Africa, the Caribbean, and Latin America, where it is a prominent source of protein in the human diet, as well as wood for fuel and light duty structural applications such as thatch for roofing [2]. Pigeonpea has now moved from an “orphan legume crop” to one of the promising pluses where genomics-assisted breeding approaches for a sustainable crop improvement are routine by Pigeonpea Genome Initiative, an effort of various researchers [3]. The first pigeonpea EST dataset provides a transcriptomic resource for gene discovery and development of functional markers associated with biotic stress resistance [4]. Root is the major part of water and nutrition uptake in pigeonpea which has a deep and extensive root system that provides access to water stored deep in the soil profile when that in the surface layer is depleted; this source of water is particularly important for long duration crops. In order to identify the associated genes in pigeonpea root tissues, a normalized cDNA library was constructed from pigeonpea root and expression analysis of the identified genes was carried out by reverse transcriptase PCR (RT-PCR) technique.

## 2. Materials and Methods

The pigeonpea genotype, namely, GRG-295 was selected to construct cDNA library and identification of expressed sequence tags (ESTs). The seeds of GRG-295 pigeonpea genotype were grown in petri dish for 15 days and irrigated with water. At the end of the 15th day, total RNA was isolated from root tissues by using the Trizol reagent (Invitrogen, Carlsbad, CA, USA), and mRNA was further isolated by using the PolyATract mRNA Isolation System (Promega, Madison, WI, USA). The quantification of RNA was verified by absorption ratio of OD_260/280_ and by formaldehyde gel electrophoresis. The first and second strands of cDNA were synthesized using Clontech SMARTer PCR cDNA Synthesis Kit. The cDNAs were purified by the MinElute PCR purification kit (Qiagen, Valencia, CA, USA) and ligated into a pGEM-T easy vector (Promega, Madison, WI, USA). Ligated plasmid DNAs were used for transformation into competent* E. coli* DH5*α* strain. Positive clones were selected on an ampicillin/IPTG/X-Gal LB plate. Plasmid DNA from positive clones were isolated by using REAL 96 plasmid isolation kit (Qiagen, Netherlands), and purified DNA was used for single-pass Sanger sequencing by using M13F/R universal sequencing primers on ABI sequencing machine 3500XL Genetic analyzer. All the ESTs were processed using VecScreen (http://www.ncbi.nlm.nih.gov/VecScreen/VecScreen.html) to remove vector and cloning oligo sequences and various contaminants to trim a high quality region. Based on the qualified sequences, the predicted amino acid sequences were used to search for similar peptide sequences to search for similar protein sequences in public database NCBI (http://www.ncbi.nlm.nih.gov) using the BLASTx search algorithm [5] by using default parameters of the program. The similarity scores between the cDNA clones and known sequences were represented by BLASTx probability *E* values. Further the ESTs were classified into different functional categories based on the knowledge of biochemistry, plant physiology, and molecular biology (http://www.MetaCyc.org/), GO (http://www.ebi.ac.uk/GOA/), and COG (http://www.ncbi.nlm.nih.gov/COG/) tools and by searching related abstracts in PubMed.

### 2.1. RT-PCR Analysis

Total root RNAs isolated from pigeonpea root tissues were used for reverse transcription polymerase chain reaction (RT-PCR) analysis. Genomic DNA contamination was removed by DNase I. First-strand cDNA was synthesized from each 2 *μ*g of total RNA sample using Clontech SMARTer PCR cDNA Synthesis Kit according to the manufacturer's protocol. The cDNAs were purified using a commercial column (Qiagen). To determine the expression of candidate genes, PCR was performed with 2 *μ*L of the first-strand cDNA template and gene-specific primer pairs. Gene-specific RT-PCR primers were designed with Primer 3.0 according to the EST sequences and were synthesized commercially. General PCR was conducted with annealing as required for the specific primer pairs (Table 1). RT-PCR experiments were repeated three times, and the PCR products were detected on 1.5% agarose gel.

## 3. Results and Discussion

Plant root systems serve a number of important functions, including anchoring the plant, absorbing water and nutrients, producing amino acids and hormones, and secreting organic acids, enzymes, and alkaloids [6]. The physiological significance of roots is belied by their relative structural simplicity as compared to other plant organs; major metabolic pathways such as photosynthesis lacking in root tissues have a stereotypical morphology that is conserved across taxa and throughout the life cycle of individuals. This combination of physiological relevance and structural simplicity has made roots obvious targets for functional genomics analyses [7]. As a major grain legume of semiarid tropics and a deep and extensive root system of pigeonpea represents an excellent source of identification of ESTs associated with their root tissues. So, the present work was focused on the study of genes associated with pigeonpea root tissues.

In the present investigation, the cDNA library was constructed in order to identify ESTs associated with pigeonpea roots and their functional analysis was carried out. The total RNA from the pigeonpea root tissue was isolated and the first and second cDNA strand was synthesized. The presence of the gene in plasmid construct of colonies was confirmed by colony PCR. The colony PCR showed that the size of these inserts ranged from 200 to 800 bp. Out of 400 bacterial clones, the plasmid construct of 250 positive recombinant clones was sequenced in single passed sequencing reaction from 3′ end using M13 forward/reverse primer and the sequence data was subjected to BLAST analysis. The leading sequences, tailing of the sequence, and poor quality sequences were excluded firstly. Finally, 105 high quality ESTs were retained which were clustered into 72 unigenes comprising 25 contigs and 47 singlets (Table 2) and were compared with NCBI nonredundant protein database using BLASTx algorithm and default parameters. In BLASTx analysis, it was shown that most of the sequences were having a significant homology with known proteins. Sequences that had no significant homology with protein database were compared to nucleotide BLAST using default parameters. The ESTs were deposited to NCBI dbEST with the accession number of JK973637 to JK973741 (Table 3).

**Figure 1:**
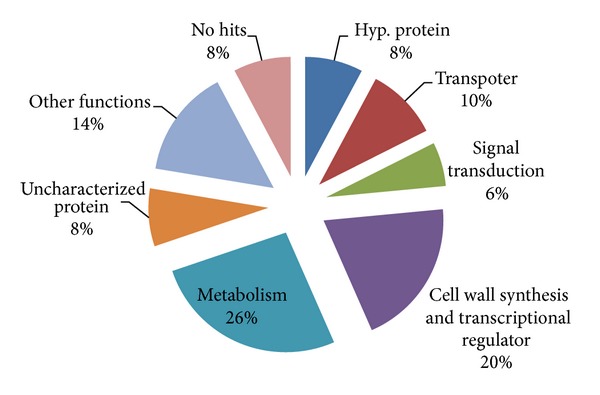
Functional classification of ESTs of pigeonpea generated by single pass sequencing.

The ESTs were categorized into 8 diverse functional group consisting of 10% transporter genes, 6% signal transduction genes, 20% cell growth and transcriptional regulator genes, and 26% metabolism genes. In other genes, 8% genes were uncharacterized, 8% hypothetical genes, 10% genes with no significant match, and 14% genes was involved in other functions. There were 8% of the ESTs found that did not show any match to known proteins in BLASTx program and that suggest novel nature of those genes (Figure 1).

**Figure 2:**
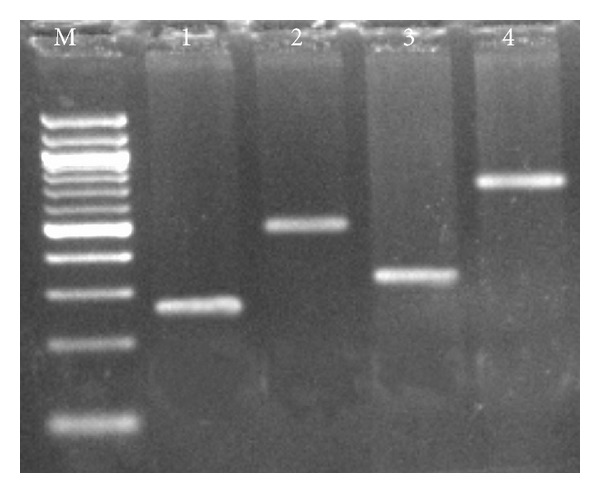
Validation of cDNA library results by RT-PCR (M-100 bp ladder, 1-*S-adenosylmethionine synthetase*; 2-*phosphoglycerate kinase*; 3-*serine carboxypeptidase*; and 4-*methionine aminopeptidase*).

In order to validate the ESTs generated from cDNA library, the expression of the four genes which were involved in different metabolic pathways was analysed by RT-PCR. RT-PCR results showed that the expression levels of four candidate genes, namely;* S-adenosylmethionine synthetase, phosphoglycerate kinase, serine carboxypeptidase,* and* methionine aminopeptidase,* were clearly expressed in pigeonpea roots (Figure 2). It was concluded that overall, there was a good agreement between the cDNA library data and the RT-PCR results.

*S-Adenosylmethionine synthetase* (SAMS) comprises of two cDNAs in* Pinus contorta* among which SAMS1 is expressed in roots and exhibits a specific expression pattern in the meristem at the onset of adventitious root development [8]. SAMS also catalyses the nucleophilic substitution reaction from between methionine and ATP into S-adenosylmethionine which have central role in several biological process in plants, namely, methyl group donor in trimethylation of lignin, DNA, and alkaloids as well as donor of aminopropyl moieties in ethylene and polyamine synthesis [8, 9].

*Phosphoglycerate kinase* superfamily has a diverse function in numerous metabolic processes like generation of precursor metabolites and energy, carbohydrate metabolism, phosphorous metabolism, glycolysis, kinase activity, ATP binding activity, and so forth. The presence of* phosphoglycerate kinase* transcripts in the cDNA library supports its diverse function in pigeonpea root.

*Serine carboxypeptidase* differentially expressed in root and other tissues is responsible for the synthesis of sinapoylcholine and sinapoylmalate in* Arabidopsis* which encodes 51 proteins annotated as* serine carboxypeptidase*-like enzymes and emerged as a new group of acyltransferase enzymes that are able to modify plant natural products [10–12].* Methionine aminopeptidase*, a ubiquitous enzyme, differentially expressed in root tissues is one of the central enzymes in protein synthesis that catalyzes N-terminal methionine from proteins [13].

A few important ESTs, namely, type 2 metallothionein, TBP-associated factor, ABC transporter, and phosphatidylcholine transfer protein, were also abundantly found in the cDNA library. Reddy et al. [14] found abundant metallothionein genes in normalized library from rice leaves and suggested that they might perform essential functions of plant growth beside metal detoxification. TATA-binding protein and TBP-associated factors are transcriptional factors which are predominantly involved in RNA polymerase II mediated transcription process [15]. ABC transporters play an important role in organ growth, plant nutrition, plant development, response to abiotic stress, and the interaction of the plant with its environment [16]. Phosphatidylcholine is usually the most abundant phospholipids in animals and plants, often amounting to almost 50% of the total, and as such it is obviously the key building block of membrane bilayers making up a very high proportion of the outer leaflet of the plasma membrane [17]. Similarly numerous ESTs like* glycine dehydrogenase*, signal proteins, root nodule extensions, membrane proteins, and **β*-glucosidase* were observed in the cDNA library constructed from pigeonpea root tissues, which is thought to play possible roles in plant metabolism, growth, and development.

Apart from the known ESTs, 6 EST transcripts (JK973668, JK973685, JK973701, JK973714, JK973715, and JK973718) were observed as not having any significant match in NCBI database. Along with that uncharacterized and hypothetical proteins were also observed in cDNA library which were thought to impart in the cardinal role in root tissues of pigeonpea.

## 4. Conclusion

Our present investigation contains a precise repertoire of transcripts associated with the various metabolic functions in pigeonpea root. These ESTs appear to be involved in multiple metabolism pathways in the plant's physiological and biochemical processes. In addition to known genes, some ESTs were unknown and uncharacterized, whose functional roles remain unclear and require further investigation in future. The root transcriptome characterized in this study markedly provides a unique resource for investigating the functional specificities of the root system. These EST tags may be useful for functional gene annotation, analysis of splice site variants and intron/exon determination, and evaluation of gene homologies or KEGG pathway confirmation.

## Figures and Tables

**Table 1:** Primer sequences of subset of ESTs for RT-PCR analysis.

Accession number	Putative function	Optimum *T*_*m*_ (°C)	Primer sequence (5′-3′)
JK973671	S-Adenosylmethionine synthetase	61	F-AGAGGAAAT CGGT GCTGGTG
			R-GCAGCAATTTGGTCGTTGGT

JK973674	Phosphoglycerate kinase	59	F-TCCCGATCCCGATACCCTAC
			R-CAGCACGCTTTTCAGCAGTT

JK973715	Serine carboxypeptidase	60	F-ACATGAAGCTCAGTGGAGGAG
			R-AGCCATGGCCTCCAATCTTC

JK973726	Methionine aminopeptidase	60	F-GGCAT TGAAAGTTGGGCAGG
			R-GATTGCAGCACCGACATCAC

**Table 2:** Summary of ESTs library.

Total clone sequenced	250
ESTs taken for analysis	105
Number of unigenes	72
Number of contigs	25
Number of singlets	47
Average length of unigenes	442 bp
Average length of ESTs	437 bp
% GC content of unigenes	50.3
% GC content of ESTs	51.2

**Table 3:** Homology search of the ESTs generated from pigeonpea root along with their *E* value using BLASTx programme.

Sl. number	Gen bank accession number	Length (in bp)	Homologous protein	*E*-value
1	JK973637	560	Vesicle-associated membrane protein 727-like [*Glycine max*]	1*e* − 111
2	JK973638	231	Mitochondrial inner membrane protein OXA1-like [*Vitis vinifera*]	5*e* − 41
3	JK973639	630	Uncharacterized protein [*Arabidopsis thaliana*]	9*e* − 159
4	JK973640	621	Hypothetical protein [*Sorghum bicolor*]	9*e* − 139
5	JK973641	570	GTP-binding protein hflx [*Medicago truncatula*]	6*e* − 13
6	JK973642	412	Unknown [*Glycine max*]	1.1
7	JK973643	264	Secretory carrier-associated membrane protein 3-like [*Vitis vinifera*]	2*e* − 55
8	JK973644	438	Histone 2 [*Populus trichocarpa*]	5*e* − 63
9	JK973645	200	RNA recognition motif-containing protein [*Arabidopsis thaliana*]	4*e* − 10
10	JK973646	870	Beta-glucosidase [*Arabidopsis thaliana*]	0.0
11	JK973647	555	Epsin N-terminal homology (ENTH) domain-containing protein [*Medicago truncatula*]	4*e* − 36
12	JK973648	210	Gamma-gliadin precursor [*Ricinus communis*]	7*e* − 10
13	JK973649	210	AP2/ERF and B3 domain-containing transcription factor [*Arabidopsis thaliana*]	4*e* − 40
14	JK973650	474	Glycine dehydrogenase (decarborcylating mitochondrial like) [*Glycine max*]	4*e* − 102
15	JK973651	432	Signal recognition particle subunit [*Arabidopsis thaliana*]	9*e* − 80
16	JK973652	227	TBP-associated factor 6B [*Arabidopsis thaliana*]	0.051
17	JK973653	290	TBP-associated factor 6B [*Arabidopsis thaliana*]	8*e* − 48
18	JK973654	303	Transcription factor [*Medicago truncatula*]	6*e* − 29
19	JK973655	432	Uncharacterized protein [*Glycine max*]	8*e* − 11
20	JK973656	237	Nuclear cap-binding protein subunit [*Arabidopsis thaliana*]	8*e* − 48
21	JK973657	501	Glucan endo-1,3-beta-glucosidase-like protein 3-like [*Glycine max*]	2*e* − 04
22	JK973658	499	DNA-binding protein RAV1 [*Zea mays*]	4*e* − 25
23	JK973659	560	Two-component response regulator ARR9 [*Vitis vinifera*]	1*e* − 45
24	JK973660	210	Ribonucleoside-diphosphate reductase small chain like [*Glycine max*]	2*e* − 34
25	JK973661	748	Hypothetical protein [*Oryza sativa*]	
26	JK973662	742	Cyclin-dependent protein kinase complex component [*Aspergillus kawachii*]	0.12
27	JK973663	406	Elongation factor 2 [*Nicotiana tobacum*]	5*e* − 39
28	JK973664	841	Expressed protein [*Oryza sativa*]	7.3
29	JK973665	385	Hypothetical protein [*Vitis vinifera*]	2.1
30	JK973666	544	ADP-ribosylation factor-like 8d [*Nicotiana tobacum*]	2*e* − 105
31	JK973667	510	Jmjc domain-containing protein 4-like [*Brachypodium distachyon*]	6*e* − 77
32	JK973668	630	**Nosignificant match**	
33	JK973669	804	Gag-pro[*Pisum sativum*]	9*e* − 18
34	JK973670	320	Hypothetical protein MTR [*Medicago truncatula*]	2*e* − 13
35	JK973671	509	Putative S-adenosylmethionine synthetase [*Capsicum annum*]	6*e* − 114
36	JK973672	371	ABC transporter F family member 1-like [*Brachypodium distachyon*]	4*e* − 60
37	JK973673	513	Predicted protein [*Hordeum vulgare*]	2*e* − 89
38	JK973674	465	Phosphoglycerate kinase, cytosolic [*Triticum aestivum*]	2*e* − 97
39	JK973675	465	Phosphoglycerate kinase, cytosolic [*Triticum aestivum*]	2*e* − 97
40	JK973676	457	Unknown [*Picea sitchensis*]	0.97
41	JK973677	785	GTP-binding signal recognition particle SRP54 [*Medicago truncatula*]	7*e* − 51
42	JK973678	241	Signal recognition particle 54 kDA subunit precursor [*Pisum sativum*]	0.044
43	JK973679	475	Protein SET-like [*Brachypodium distachyon*]	7*e* − 33
44	JK973680	350	EIN3-binding F-box protein [*Brachypodium distachyon*]	8*e* − 65
45	JK973681	389	Predicted protein [*Hordeum vulgare*]	4*e* − 27
46	JK973682	272	40S ribosomal protein S24-2-like [*Brachypodium distachyon*]	2*e* − 57
47	JK973683	210	Putative calcium exchanger [*Triticum dicoccoides*]	4*e* − 27
48	JK973684	478	Thioredoxin [*Medicago truncatula*]	2*e* − 75
49	JK973685	210	**Nosignificant match**	
50	JK973686	553	Root nodule extension [*Pisum sativa*]	5*e* − 04
51	JK973687	600	Signal recognition particle protein [*Oryza sativa*]	5*e* − 08
52	JK973688	544	ADP-ribosylation factor-like 8d [*Nicotiana tobacum*]	2*e* − 105
53	JK973689	395	MLO-like protein [*Medicago truncatula*]	2*e* − 24
54	JK973690	250	Phosphatidylcholine transfer protein [*Ricinus communis*]	3*e* − 09
55	JK973691	215	Phosphatidylcholine transfer protein [*Ricinus communis*]	3*e* − 09
56	JK973692	411	Unknown [*Glycine max*]	1.1
57	JK973693	246	Phosphatidylcholine transfer protein [*Ricinus communis*]	5*e* − 09
58	JK973694	246	Phosphatidylcholine transfer protein [*Ricinus communis*]	5*e* − 09
59	JK973695	420	Uncharacterized protein [*Zea mays*]	9*e* − 83
60	JK973696	350	Uncharacterized protein [*Zea mays*]	5*e* − 51
61	JK973697	244	Jmjc domain-containing protein 4-like [*Brachypodium distachyon*]	5*e* − 27
62	JK973698	415	Os01g0678900 [*Oryza sativa*]	8*e* − 11
63	JK973699	282	D-3-Phosphoglycerate dehydrogenase [*Ricinus communis*]	3*e* − 51
64	JK973700	558	60S ribosomal protein [*Vitis vinifera*]	8*e* − 65
65	JK973701	490	**Nosignificant match**	
66	JK973702	556	Root nodule extension [*Pisum sativum*]	3*e* − 07
67	JK973703	313	Coiled-coil domain-containing protein [*Ricinus communis*]	2*e* − 65
68	JK973704	311	Coiled-coil domain-containing protein [*Ricinus communis*]	1*e* − 64
69	JK973705	320	Hypothetical protein MTR_4g076190 [*Medicago truncatula*]	2*e* − 13
70	JK973706	509	Putative S-adenosylmethionine synthetase [*Capsicum annuum*]	9*e* − 119
71	JK973707	460	Transmembrane emp24 domain-containing protein 10-like [*Brachypodium distachyon*]	2*e* − 45
72	JK973708	482	60S ribosomal protein [*Zea mays*]	3*e* − 52
73	JK973709	395	MLO5-like protein [*Medicago truncatula*]	2*e* − 24
74	JK973710	480	Type 2 metallothionein [*Prosopis * *juliflora*]	4*e* − 28
75	JK973711	556	Root nodule extension [*Pisum sativa*]	3*e* − 07
76	JK973712	174	Hypothetical protein [*Oryza sativa*]	0.86
77	JK973713	278	**Nosignificant match**	
78	JK973714	439	**Nosignificant match**	
79	JK973715	439	Serine carboxypeptidase-like 19-like [*Glycine max*]	2*e* − 31
80	JK973716	551	Serine carboxypeptidase-like 19-like [*Glycine max*]	9*e* − 39
81	JK973717	347	**Nosignificant match**	
82	JK973718	661	Gamma-glutamyl hydrolase [*Vitis vinifera*]	1*e* − 54
83	JK973719	499	Type 2 metallothionein [*Prosopis juliflora*]	2*e* − 30
84	JK973720	463	Nodulin mtn21/eama-like transporter protein [*Arabidopsis thaliana*]	2*e* − 44
85	JK973721	200	RNA recognition motif-containing protein [*Arabidopsis thaliana*]	4*e* − 10
86	JK973722	280	Beta-glucosidase 44-like [*Glycine max*]	3*e* − 52
87	JK973723	424	V-type proton ATpase 21 kDA proteolipid subunit [*Medicago truncatula*]	2*e* − 29
88	JK973724	210	AP2/ERF and B3 domain-containing transcription factor [*Arabidopsis thaliana*]	4*e* − 40
89	JK973725	540	Polyribonucleotide nucleotidyltransferase [*Vitis vinifera*]	3*e* − 07
90	JK973726	649	Methionine aminopeptidase 2B-like [*Brachypodium distachyon*]	3*e* − 40
91	JK973727	585	Hypothetical protein [*Vitisvinifera*]	6.9
92	JK973728	487	Unknown [*Lotus japonica*]	1
93	JK973729	558	60S ribosomal protein [*Vitisvinifera*]	8*e* − 65
94	JK973730	314	D-3-Phosphoglycerate dehydrogenase [*Ricinus communis*]	1*e* − 55
95	JK973731	350	EIN3-binding F-box protein 1-like [*Brachypodium distachyon*]	8*e* − 65
96	JK973732	499	DNA-binding protein RAV1 [*Zea mays*]	4*e* − 25
97	JK973733	239	NOT2/NOT3/NOT5 family protein [*Oryza sativa*]	3*e* − 62
98	JK973734	272	40S ribosomal protein S24-2-like [*Brachypodium distachyon*]	2*e* − 57
99	JK973735	559	Signal recognition particle subunit [*Arabidopsis thaliana*]	9*e* − 80
100	JK973736	793	Vesicle-associated membrane protein 727-like [*Glycine max*]	1*e* − 111
101	JK973737	649	Methionine aminopeptidase 2B [*Brachypodium distachyon*]	1*e* − 24
102	JK973738	210	Gamma-gliadin precursor [*Ricinus communis*]	7*e* − 10
103	JK973739	553	Unknown protein product [*Glycine max*]	1*e* − 04
104	JK973740	600	Glycine dehydrogenase [*Glycine max*]	4*e* − 102
105	JK973741	572	Glucan endo-1,3-beta-glucosidase-like protein 3-like [*Glycine max*]	2*e* − 04

## Abbreviations

ESTs: Expressed sequence tags
RT PCR: Reverse transcription polymerase chain reaction
SAMS: S-Adenosyl methionine synthetase.
